# Decoding motor plans using a closed-loop ultrasonic brain–machine interface

**DOI:** 10.1038/s41593-023-01500-7

**Published:** 2023-11-30

**Authors:** Whitney S. Griggs, Sumner L. Norman, Thomas Deffieux, Florian Segura, Bruno-Félix Osmanski, Geeling Chau, Vasileios Christopoulos, Charles Liu, Mickael Tanter, Mikhail G. Shapiro, Richard A. Andersen

**Affiliations:** 1 Division of Biology and Biological Engineering, California Institute of Technology, Pasadena, CA USA; 2 David Geffen School of Medicine at UCLA, Los Angeles, CA USA; 3 Physics for Medicine Paris, INSERM, CNRS, ESPCI Paris, PSL Research University, Paris, France; 4 INSERM Technology Research Accelerator in Biomedical Ultrasound, Paris, France; 5 Iconeus, Paris, France; 6 T&C Chen Brain-Machine Interface Center, California Institute of Technology, Pasadena, CA USA; 7 Department of Bioengineering, University of California, Riverside, Riverside, CA USA; 8 Department of Neurological Surgery, Keck School of Medicine of USC, Los Angeles, CA USA; 9 USC Neurorestoration Center, Keck School of Medicine of USC, Los Angeles, CA USA; 10 Rancho Los Amigos National Rehabilitation Center, Downey, CA USA; 11 Division of Chemistry & Chemical Engineering, California Institute of Technology, Pasadena, CA USA; 12 Andrew and Peggy Cherng Department of Medical Engineering, California Institute of Technology, Pasadena, CA USA; 13 Howard Hughes Medical Institute, Pasadena, CA USA

**Keywords:** Brain-machine interface, Ultrasound, Neural decoding, Motor control

## Abstract

Brain–machine interfaces (BMIs) enable people living with chronic paralysis to control computers, robots and more with nothing but thought. Existing BMIs have trade-offs across invasiveness, performance, spatial coverage and spatiotemporal resolution. Functional ultrasound (fUS) neuroimaging is an emerging technology that balances these attributes and may complement existing BMI recording technologies. In this study, we use fUS to demonstrate a successful implementation of a closed-loop ultrasonic BMI. We streamed fUS data from the posterior parietal cortex of two rhesus macaque monkeys while they performed eye and hand movements. After training, the monkeys controlled up to eight movement directions using the BMI. We also developed a method for pretraining the BMI using data from previous sessions. This enabled immediate control on subsequent days, even those that occurred months apart, without requiring extensive recalibration. These findings establish the feasibility of ultrasonic BMIs, paving the way for a new class of less-invasive (epidural) interfaces that generalize across extended time periods and promise to restore function to people with neurological impairments.

## Abstract (web summary)

Griggs, Norman et al. demonstrate a closed-loop ultrasound-based brain–machine interface (BMI) in rhesus macaques. The ultrasound BMI is capable of controlling eight independent movement directions and is stable across months without retraining.

## Main

**Extended Data Fig. 1:**
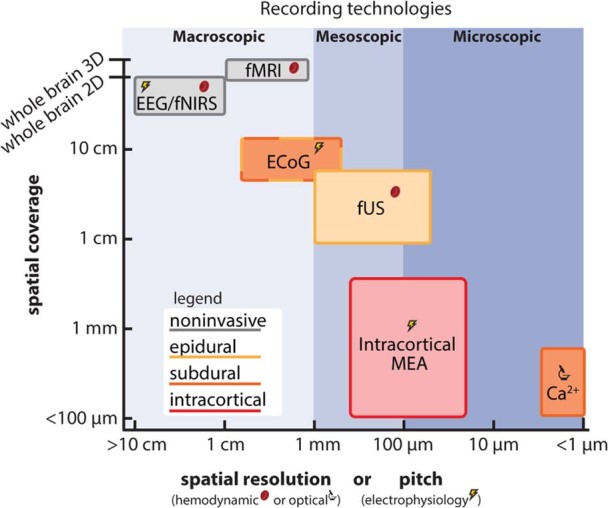
Functional ultrasound imaging can record mesoscopic neural populations with good spatial coverage. Comparison of spatial coverage, invasiveness, and spatial resolution between different large animal recording technologies. Spatial coverage refers to maximum dimension of brain volume sampling. MEA: multi-electrode array; Ca^2+^: calcium imaging; ECoG: electrocorticography; EEG: electroencephalogram; fNIRS: functional near-infrared spectroscopy; fMRI: functional magnetic resonance imaging; fUS: functional ultrasound imaging.

Brain–machine interfaces (BMIs) translate complex brain signals into computer commands and are a promising method to restore the capabilities of human patients with paralysis^1^. Numerous methods have been used to record brain signals for these BMIs, including intracortical multielectrode arrays (MEAs), electrocorticography (ECoG), functional near-infrared spectroscopy (fNIRS), electroencephalography (EEG) and functional magnetic resonance imaging (fMRI) (Extended Data Fig. 1). These methods have various trade-offs between performance, invasiveness, spatial coverage, spatiotemporal resolution, portability and decoder stability across sessions (Supplementary Table 1). For example, intracortical MEAs have been used to decode up to 62 words per minute^2^ and control a robotic arm^3^, but each array can only sample from a small area of cortex (∼4 × ∼4 mm) located on gyral crowns. Conversely, fMRI is noninvasive and samples from the entire brain, however, fMRI-based BMIs have only been demonstrated to decode approximately 1 char per min^4^ or control up to four movement directions^5^.

**Extended Data Fig. 2:**
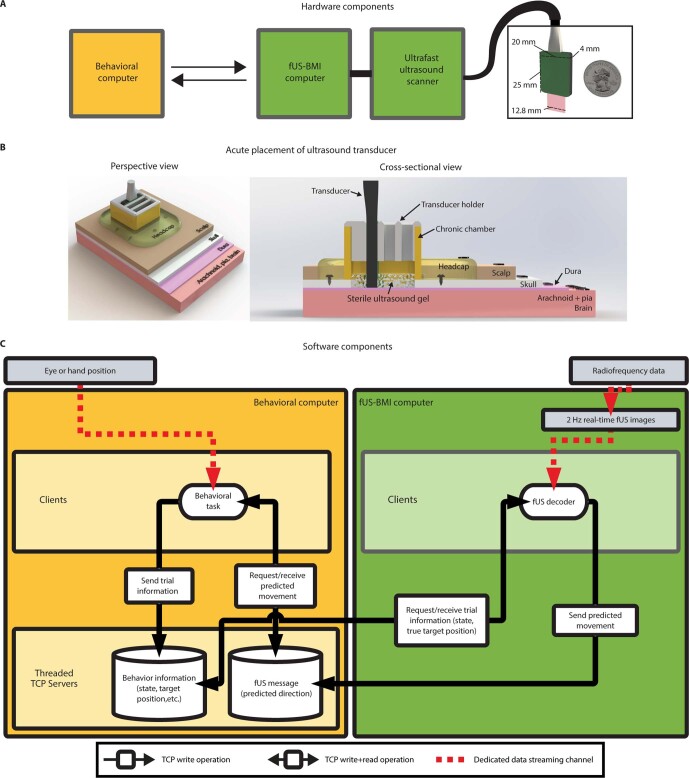
Hardware and software for real-time fUS-BMI. **(a)** Hardware components. The behavioral computer communicates via TCP with the fUS-BMI computer. The fUS-BMI computer is attached via a dedicated data cable to the ultrafast ultrasound scanner. The fUS-BMI computer receives the radiofrequency data from the ultrasound scanner and performs the real-time 2 Hz Power Doppler image formation. The ultrafast ultrasound scanner is attached to and controls a 15.6 MHz ultrasound transducer. **(b)** Acute placement of ultrasound transducer. The ultrasound transducer is held by a custom 3D-printed slotted plug that fits in the monkey chamber. This chamber is chronic and is embedded in a headcap attached to the monkey skull. The acute ultrasound transducer rests above the dura along with sterile ultrasound coupling gel. **(c)** Software components. A threaded TCP server in Python 2.7 received, parsed, and sent information between the computer running the PsychoPy behavior software and the real-time fUS-BMI computer. Upon queries from the fUS-BMI computer (‘fUS decoder’), this server transferred necessary task information. The client-server architecture was specifically designed to prevent data leaks, that is, the actual movement direction was never transmitted to the fUS-BMI until after a successful trial had ended. The TCP server also received the fUS-BMI prediction and passed it to the PsychoPy software when queried. The average server write-read-parse time was 31 +/− 1 (mean ± STD) ms during offline testing between two Windows computers on a local area network.

Functional ultrasound (fUS) imaging is a recently developed technology that is poised to enable a new class of epidural BMIs that can record from large regions of the brain and decode spatiotemporally precise patterns of activity. fUS neuroimaging uses ultrafast pulse-echo imaging to simultaneously sense changes in cerebral blood volume (CBV) from multiple brain regions^6^. These CBV changes are well correlated with single-neuron activity and local field potentials^7,8^. It has a high sensitivity to slow blood flow (∼1 mm s^−1^ velocity) and balances good spatiotemporal resolution (100 μm; <1 s) with a large and deep field of view (∼2 cm; Extended Data Fig. 1). fUS can successfully image through the dura and multiple millimeters of granulation tissue^9^ (Extended Data Fig. 2a). However, fUS imaging currently requires either a cranial opening or an acoustic window^10^ in large animals because the ultrasound signal is severely attenuated by bone^11^.

Previously, we demonstrated that fUS neuroimaging possesses the sensitivity and field-of-view to decode movement intention on a single-trial basis simultaneously for two directions (left/right), two effectors (hand/eye) and task state (go/no-go)^9^. However, we performed this post hoc (offline) analysis using prerecorded data. In this study, we demonstrate an online, closed-loop functional ultrasound brain–machine interface (fUS-BMI). In addition, we present key advances that build on previous fUS neuroimaging studies, including decoding eight movement directions and designing decoders stable across >40 days.

## Results

**Fig. 1:**
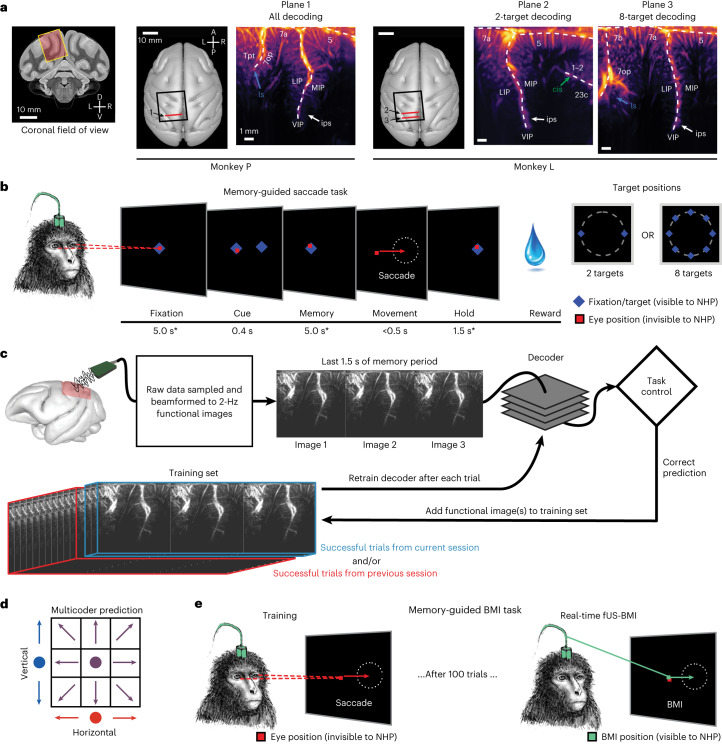
Anatomical recording planes and behavioral tasks. **a**, Coronal fUS imaging planes used for monkeys P and L. The approximate fUS field of view superimposed on a coronal MRI slice. The recording chambers were placed surface normal to the skull above a craniectomy (black square). The ultrasound transducer was positioned to acquire a consistent coronal plane across different sessions (red line). The vascular maps show the mean power Doppler image from a single imaging session. Different brain regions are labeled in white text, and the labeled arrows point to brain sulci. D, dorsal; V, ventral; L, left; R, right; A, anterior; P, posterior; ls, lateral sulcus; ips, intraparietal sulcus; cis, cingulate sulcus. Anatomical labels are based upon ref. ^63^. **b**, Memory-guided saccade task. * ±1,000 ms of jitter for fixation and memory periods; ±500 ms of jitter for hold period. The peripheral cue was chosen from two or eight possible target locations depending on the specific experiment. Red square, monkey’s eye position (not visible to the monkey). NHP, nonhuman primate, that is, monkey. **c**, fUS-BMI algorithm. Real-time 2-Hz functional images were streamed to a linear decoder that controlled the behavioral task. The decoder used the last three fUS images of the memory period to make its prediction. If the prediction was correct, the data from that prediction were added to the training set. The decoder was retrained after every successful trial. The training set consisted of trials from the current session and/or from a previous fUS-BMI session. **d**, Multicoder algorithm. For predicting eight movement directions, the vertical component (blue) and the horizontal component (red) were separately predicted and then combined to form each fUS-BMI prediction (purple). **e**, Memory-guided BMI task. The BMI task is the same as in **b** except that the movement period is controlled by the brain activity (via fUS-BMI) rather than eye movements. After 100 successful eye movement trials, the fUS-BMI controlled the movement prediction (closed-loop control). During the closed-loop mode, the monkey had to maintain fixation on the center fixation cue until reward delivery. Red square, monkey’s eye position (not visible to the monkey); green square, BMI-controlled cursor (visible to the monkey).

We used a miniaturized 15.6 MHz ultrasound transducer paired with a real-time ultrafast ultrasound acquisition system to stream 2 Hz fUS images from two monkeys as they performed memory-guided eye movements (Fig. 1 and Extended Data Fig. 2a). Before the experiments, we performed a craniectomy over the left posterior parietal cortex (PPC) in both monkeys. During each experiment session (*n* = 24 sessions; Extended Data Table 1), we positioned the transducer surface normal to the brain above the dura mater (Fig. 1a and Extended Data Fig. 2b) and recorded from coronal planes of the left PPC, a sensorimotor association area important for goal-directed movements and attention^12^. This technique achieved a large field of view (12.8-mm width, 16-mm depth, ∼400-μm plane thickness) while maintaining high spatial resolution (100 μm × 100 μm in-plane). This allowed us to stream high-resolution hemodynamic changes across multiple PPC regions simultaneously, including the lateral (LIP) and medial (MIP) intraparietal cortex (Fig. 1a). The LIP and MIP are involved in planning eye and reach movements respectively^9,13,14^, making the PPC a good region from which to record effector-specific movement signals.

We streamed real-time fUS images into a BMI decoder that used principal component (PCA) and linear discriminant analysis (LDA) to predict planned movement directions. The BMI output then directly controlled the behavioral task (Fig. 1c). To build the initial training set for the decoder, each monkey initially performed instructed eye movements to a randomized set of two or eight peripheral targets. We used the fUS activity during the delay period preceding successful eye movements to train the decoder. During this initial training phase, successful trials were defined as the monkey performing the eye movement to the correct target and receiving the liquid reward. After 100 successful training trials, we switched to the closed-loop BMI mode where the intended movement came from the fUS-BMI (Fig. 1e). During this closed-loop BMI mode, the monkey continued to fixate on the center cue until reward delivery. During the interval between a successful trial and the subsequent trial, we retrained the decoder, continuously updating the decoder model as each monkey used the fUS-BMI. During the closed-loop fUS-BMI mode, successful trials were defined as a correct prediction plus the monkey maintaining fixation on the center cue until reward delivery.

### Online decoding of two eye-movement directions

**Fig. 2:**
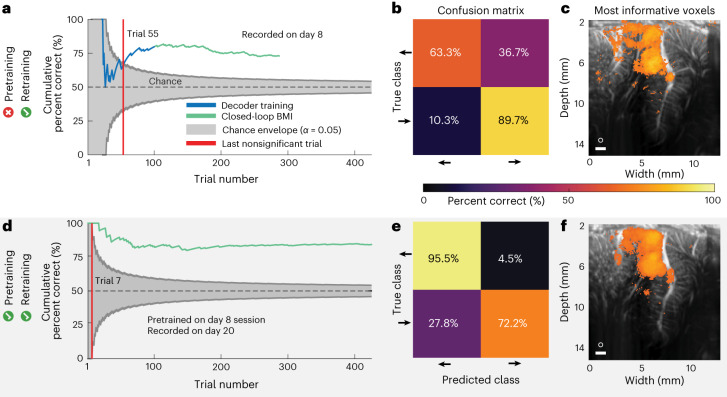
Example sessions decoding two saccade directions (monkey P). **a**, Cumulative decoding accuracy as a function of trial number. Blue represents fUS-BMI training where the monkey controlled the task using overt eye movements. The BMI performance shown in blue was generated post hoc with no impact on the real-time behavior. Green represents trials under fUS-BMI control where the monkey maintained fixation on the center cue and the movement direction was controlled by the fUS-BMI. Gray chance envelope, 90% binomial distribution; red line, last nonsignificant trial. **b**, Confusion matrix of final decoding accuracy across the entire session represented as a percentage (rows add to 100%). **c**, Searchlight analysis represents the 10% voxels with the highest decoding accuracy (threshold is *q* *≤* 1.66×10^−6^). White circle, 200-μm searchlight radius. Scale bar, 1 mm. **d**–**f**, Same format as in **a**–**c**. fUS-BMI was pretrained using data collected from a previous session. **d**, Cumulative decoding accuracy as a function of trial number. **e**, Confusion matrix of final decoding accuracy. **f**, Searchlight analysis represents the 10% of voxels with the highest decoding accuracy (threshold is *q* *≤* 2.07 × 10^−13^). White circle, 200-μm searchlight radius. Scale bar, 1 mm.

To demonstrate feasibility of an fUS-BMI, we first performed online, closed-loop decoding of two movement directions (Fig. 2). Each monkey initially performed 100 successful memory-guided saccades to the cued left or right target (Fig. 1b) while we streamed fUS images from the left PPC. After 100 trials, we switched to closed-loop decoding where the monkey now controlled the task direction using his movement intention, that is, the brain activity detected by the fUS-BMI in the last three fUS images of the memory period (Fig. 1c,e). At the conclusion of each closed-loop decoding trial, the monkey received visual feedback about the fUS-BMI prediction. We added the fUS images from each successful trial to our training set and retrained the decoder after each trial (Fig. 1c). We assessed the decoder’s performance throughout the training (20–100 trials; blue line) and closed-loop decoding (101+ trials; green line) using cumulative percent correct (Fig. 2a). During the initial training period (20–100 trials), the decoder’s prediction was not visible to the monkey, that is, no green dot was shown until the closed-loop decoding began after trial 100.

In the second closed-loop two-direction session, the decoder reached significant accuracy (*P* < 0.05; one-sided binomial test) after 55 training trials and improved in accuracy until peaking at 82% accuracy at trial 114 (Fig. 2a). The decoder predicted both directions well above chance level but displayed better performance for rightward movements (Fig. 2b). To understand which brain regions were most important for the decoder performance, we performed a searchlight analysis with a 200 μm, that is, 2 voxel, radius (Fig. 2c). Dorsal LIP and area 7a contained the voxels most informative for decoding intended movement direction.

**Extended Data Fig. 3:**
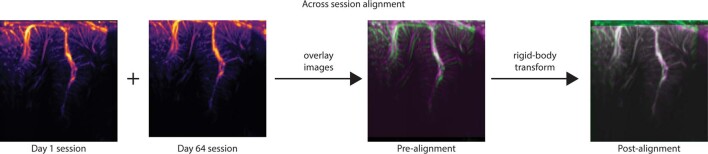
Across session alignment algorithm. We used semi-automated intensity-based rigid-body alignment to find the transform from the previous session to the new imaging plane. The alignment error is shown in the overlay where green represents the old session (Day 1) and magenta represents the new session (Day 64).

An ideal BMI needs very little training data and no retraining between sessions. BMIs using intracortical electrodes typically require recalibration for each subsequent session due to nonstationarities across days, including from difficulty recording the same neurons across multiple days^15,16^. Thanks to its wide field of view, fUS neuroimaging can image from the same brain regions over time, and therefore may be an ideal technique for stable decoding across many sessions. The neuron population identification problem is also present with ultrasound imaging, including from brain shifts relative to the ultrasound transducer between sessions. To test our hypothesis that we would have stable decoding across many sessions, we pretrained the fUS-BMI using a previous session’s data and then tested the decoder in an online, closed-loop experiment. To perform this pretraining, we first aligned the data from the previous session’s imaging plane to the current session’s imaging plane (Extended Data Fig. 3). This addressed the neuron population identification problem by allowing us to track the same neurovascular populations across different sessions. We used semiautomated rigid-body alignment to find the transform between the previous and current imaging plane, applied this two-dimensional (2D) image transform to each frame of the previous session and saved the aligned data. This semiautomated alignment process took <1 min. After we performed this image alignment, the fUS-BMI automatically loaded this aligned dataset and pretrained the initial decoder. As in the models without pretraining, we continued to use real-time retraining to incorporate the most recent successful trials. This adaptive retraining of the BMI after each successful trial allowed the BMI to incorporate session-specific changes (behavioral, anatomical, neurovascular and so on) and may allow the BMI to achieve better performance. The fUS-BMI reached significant performance substantially faster (Fig. 2d) when we used pretraining. The fUS-BMI achieved significant accuracy at Trial 7, approximately 15 min faster than the example session without pretraining.

**Fig. 3:**
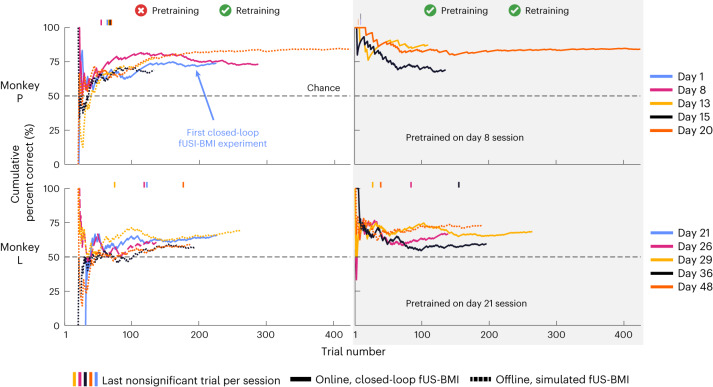
Performance across sessions for decoding two saccade directions. Cumulative decoder accuracy during each session for monkey P and L. Solid lines are real-time results and fine-dashed lines are simulated sessions from post hoc analysis of real-time fUS imaging data. Vertical marks above each plot represent the last nonsignificant trial for each session. Day number is relative to the first fUS-BMI experiment. Coarse-dashed horizontal black line represents chance performance.

To quantify the benefits of pretraining upon fUS-BMI training time and performance, we compared fUS-BMI performance across all sessions when (1) using only data from the current session, versus (2) pretraining with data from a previous session (Fig. 3). For all real-time sessions that used pretraining, we also created a post hoc (offline) simulation of the fUS-BMI results without using pretraining. For these simulated sessions without pretraining, the recorded data passed through the same classification algorithm used for the real-time fUS-BMI but did not use any data from a previous session.

#### Using only data from the current session

The cumulative decoding accuracy reached significance (*P* < 0.05; one-sided binomial test) at the end of each online, closed-loop recording session (2 of 2 sessions, monkey P; 1 of 1 session, monkey L) and most offline, simulated recording sessions (3 of 3 sessions, monkey P, 3 of 4 sessions, monkey L) (Fig. 3, left). For monkey P, decoder accuracies reached 75.43 ± 2.56% correct (mean ± s.e.m.) and took 40.20 ± 2.76 trials to reach significance. For monkey L, decoder accuracies reached 62.30 ± 2.32% correct and took 103.40 ± 23.63 trials to reach significance.

#### Pretraining with data from a previous session

**Extended Data Fig. 4:**
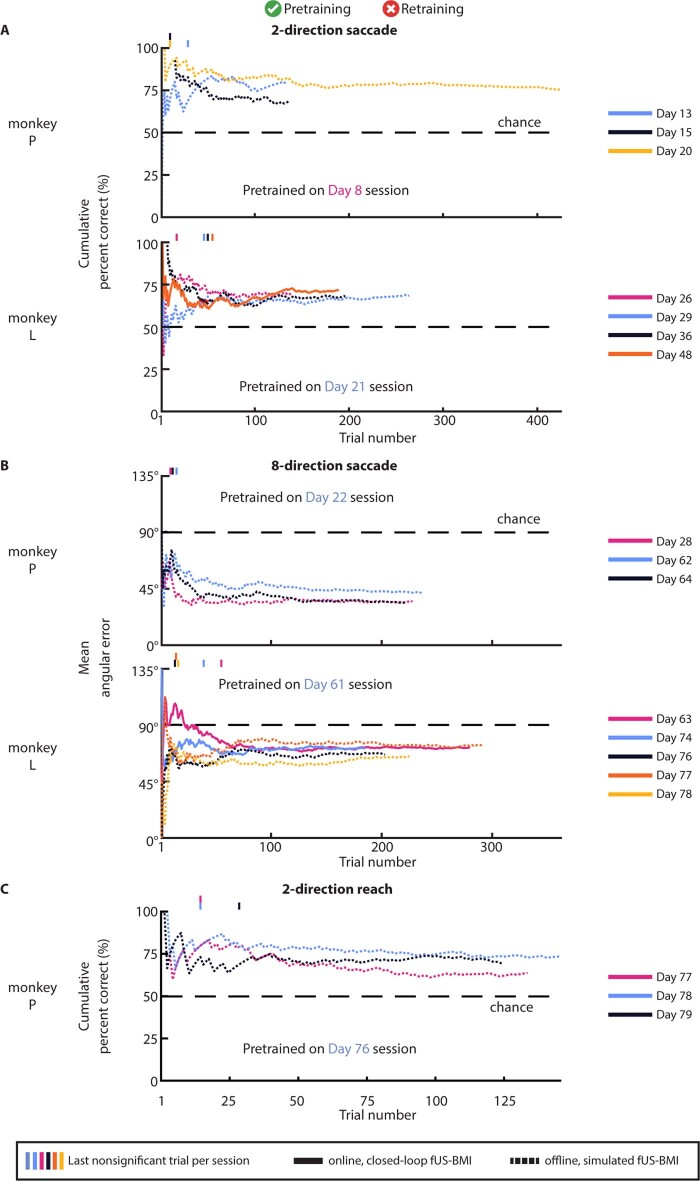
Closed-loop, real-time decoding of movement directions using pretrained model only. **(a)** Performance for 2-direction saccade decoding using only the pretrained model. Same format as in Fig. 3. **(b)** Performance for 8-direction saccade decoding using only the pretrained model. Same format as in Fig. 5. **(c)** Performance for 2-direction reach decoding using only the pretrained model. Same format as in Fig. 7.

The cumulative decoding accuracy reached significance at the end of each online, closed-loop recording session (3 of 3 sessions for monkey P, 4 of 4 sessions for monkey L) (Fig. 3, right). Using previous data reduced the time to achieve significant performance (100% of sessions reached significance sooner: monkey P, 36–43 trials faster; monkey L, 15–118 trials faster). The performance at the end of the session was not statistically different from performance in the same sessions without pretraining (paired *t*-test, *P* < 0.05). For monkey P, accuracies reached 80.21 ± 5.61% correct and took 9 ± 1 trials to reach significance. For monkey L, accuracies reached 66.78 ± 2.79% correct and took 71.00 ± 28.93 trials to reach significance. Assuming no missed trials, pretraining decoders shortened training by 10–45 min. We also simulated the effects of not using any training data from the current session, that is, using only the pretrained model (Extended Data Fig. 4a). We did not observe a statistically significant difference between the performance (final accuracy or number of trials to significant performance) for either monkey, whether current session training data were included or not.

These results demonstrate three things: (1) we can online decode two directions of movement intention from fUS signals, (2) monkeys learned to control the task using the fUS-BMI and (3) pretraining using previous session’s data greatly reduced, or even eliminated, the amount of new training data required in a new session.

### Online decoding of eight eye movement directions

**Fig. 4:**
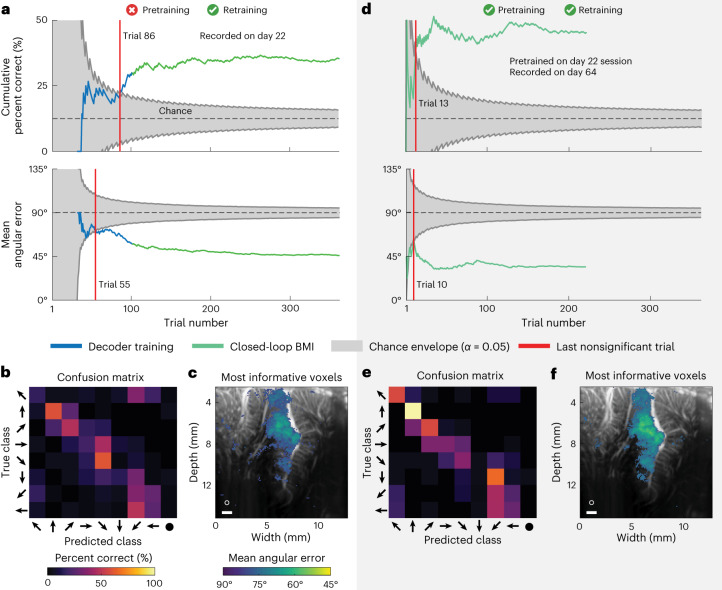
Example sessions with monkey P decoding eight saccade directions. **a**, Cumulative decoding accuracy and mean angular error as a function of trial number. Blue represents fUS-BMI training where the monkey controlled the task using overt eye movements. The BMI performance shown here was generated post hoc and tested on each new trial with no impact on the real-time behavior. Green represents trials under fUS-BMI control where the monkey maintained fixation on the center cue and the movement task direction was controlled by the fUS-BMI. Gray chance envelope, 90% binomial or permutation test distribution; red line, last nonsignificant trial. **b**, Confusion matrix of final decoding accuracy across the entire session represented as percentage (rows add to 100%). The horizontal axis plots the predicted movement (predicted class) and the vertical axis the matching directional cue (true class). **c**, Searchlight analysis represents the 10% of voxels with the lowest mean angular error (threshold is *q* *≤* 2.98 × 10^−3^). White circle, 200-μm searchlight radius. Scale bar, 1 mm. **d**–**f**, Same format as in **a**–**c**. fUS-BMI was pretrained on data from day 22 and updated after each successful trial. **d**, Cumulative decoding accuracy and mean angular error as function of trial number. **e**, Confusion matrix of final decoding accuracy across the entire session represented as percentage. **f**, Searchlight analysis represents the 10% of voxels with the lowest mean angular error (threshold is *q* *≤* 8 × 10^−5^).

Having demonstrated that we could achieve similar, but online and closed-loop, performance to our previous offline decoding study^9^, we extended the capabilities of our fUS-BMI by decoding eight movement directions in real time (Fig. 4). We used a ‘multicoder’ architecture where we predicted the vertical (up, middle or down) and horizontal (left, middle or right) components of intended movement separately and then combined those independent predictions to form a final prediction (for example, up and to the right) (Fig. 1d). This multicoder architecture allowed the decoder to incorporate our prior knowledge that PPC neurons have similar responses to neighboring movement directions but different responses to movement directions with a greater angular separation^17^. In other words, this multicoder approach incorporated the neural representational similarity between neighboring directions rather than treating the eight directions as eight independent classes.

In the first eight-direction experiment, the decoder reached significant accuracy (*P* < 0.05; one-sided binomial test) after 86 training trials and improved until plateauing at 34–37% accuracy (Fig. 4a, upper plot), compared to 12.5% chance level, with most errors indicating directions neighboring the cued direction (Fig. 4b). To quantify the proximity of each prediction to the true direction, we examined the mean angular error. The fUS-BMI reached significance at 55 trials and steadily decreased its mean angular error to 45° by the end of the session (Fig. 4a, bottom plot). Compared to the most informative voxels for the two-target eye decoder, a larger portion of LIP, including ventral LIP, contained the most informative voxels for decoding eight movement directions (Fig. 4c).

We next tested whether pretraining would aid the eight-target decoding similarly to the two-target decoding. As with the model without pretraining, we retrained the fUS-BMI in real time following each successful prediction during the intertrial interval. As before, pretraining improved the number of trials required to reach significant decoding (Fig. 4d). The fUS-BMI reached significant accuracy at trial 13, approximately 25 min earlier than using only data from the current session. The cumulative decoder accuracy reached 45% correct with a final mean angular error of 34°, which was better than the performance achieved in the example session without pretraining. The searchlight analysis indicated the same regions within LIP provided the most informative voxels for decoding (Fig. 4f) for both the example sessions with and without pretraining. Notably, we pretrained the fUS-BMI on data from 42 days before the current session. This demonstrates that fUS-BMI can remain stable over at least 42 days. It further demonstrates that we can consistently locate the same imaging plane and that mesoscopic PPC populations consistently encode for the same directions on the time span of >1 month.

**Fig. 5:**
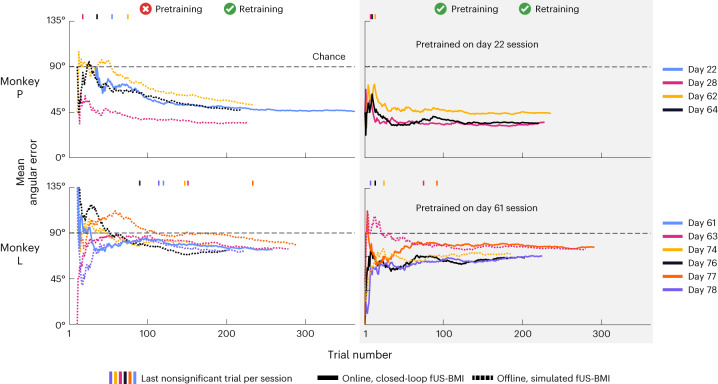
Performance across sessions for decoding eight saccade directions. Mean angular error during each session for monkey P and L. Solid lines are real-time results and fine-dashed lines are simulated sessions from post hoc analyses of real-time fUS data. Vertical marks represent the last nonsignificant trial for each session. Day number is relative to the first fUS-BMI experiment. Coarse-dashed horizontal black line represents chance performance.

#### Using only data from the current session

The cumulative decoder accuracy reached significance by the end of all real-time (2 of 2) and simulated (8 of 8) sessions (Fig. 5, left). The mean angular error for monkey P reached 45.26 ± 3.44° and the fUS-BMI took 30.75 ± 12.11 trials to reach significance. The mean angular error for monkey L reached 75.06 ± 1.15° and the fUS-BMI took 132.33 ± 20.33 trials to reach significance.

#### Pretraining with data from a previous session

The cumulative decoder accuracy reached significance by the end of all real-time (6 of 6) and simulated (2 of 2) sessions (Fig. 5, right). The fUS-BMI reached significant decoding earlier for most sessions compared to simulated post hoc data: 5 of 5 faster than monkey L; 2 of 3 faster than monkey P (third session reached significance equally fast). For monkey P, the pretrained decoders reached significance 0–51 trials faster and for monkey L, the pretrained decoders reached significance 66–132 trials faster. For most sessions, this would shorten training by up to 45 min. The performance at the end of each session was not statistically different from performance in the same session without pretraining (paired *t*-test, *P* < 0.05). The mean angular error for monkey P reached 37.82° ± 2.86° and the fUS-BMI took 10.67 ± 1.76 trials to reach significance. The mean angular error for monkey L reached 71.04° ± 2.29° and the fUS-BMI took 42.80 ± 17.05 trials to reach significance. We also simulated the effects of not using any training data from the current session, that is, using only the pretrained model (Extended Data Fig. 4b). We did not observe a statistically significant difference between the performance (final accuracy, final mean angular error or number of trials to significant performance) for either monkey, whether current session training data were included or not.

These results demonstrate online decoding of fUS signals into eight directions of movement intention, a substantial advance over decoding only contra- and ipsilateral movements. These results also show that the directional encoding within PPC mesoscopic populations is stable across >1 month, thus allowing us to reduce, or even eliminate, the need for new training data.

### Online decoding of two hand-movement directions

**Fig. 6:**
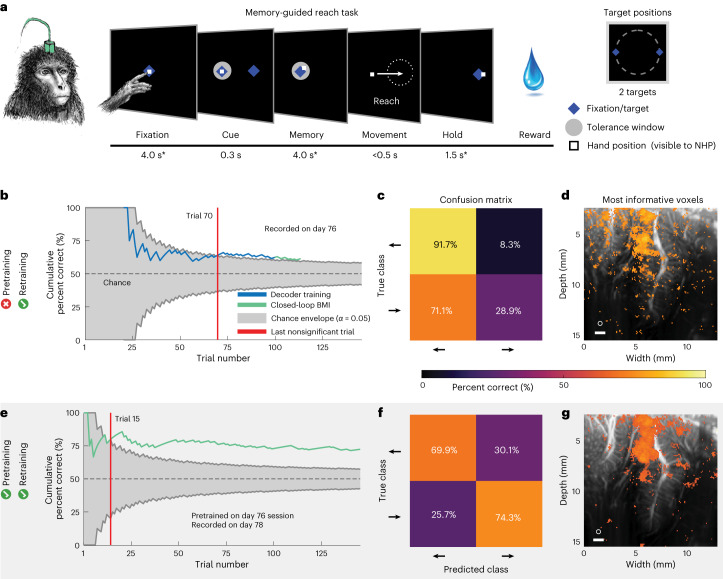
Example sessions decoding two reach directions (monkey P). **a**, Memory-guided reach task for Monkey P. Identical to memory-guided saccade task in Fig. 1b with all fixation or eye movements being replaced by maintaining touch on the screen and reach movements, respectively. * ±1,000 ms of jitter for fixation and memory periods; ±500 ms of jitter for hold period. Peripheral cue was chosen from one of two peripheral targets. The white square represents the monkey’s hand position and is visible to the monkey. **b**–**d**, Example results from session trained on data from the current session only. Same format as Fig. 2a–c. **b**, Cumulative decoding accuracy as a function of trial number. **c**, Confusion matrix of final decoding accuracy across entire session represented as percentage. **d**, Searchlight analysis represents the 10% voxels with the highest decoding accuracy (threshold is *q* *≤* 3.05 × 10^−3^). **e**–**g**, Example results from the day 78 session pretrained on data from day 76 and retrained after each successful trial. Same format as Fig. 2d–f. **e**, Cumulative decoding accuracy as a function of trial number. **f**, Confusion matrix of final decoding accuracy across entire session represented as percentage. **g**, Searchlight analysis represents the 10% voxels with the highest decoding accuracy (threshold is *q* *≤* 6.47 × 10^−3^).

Another strength of fUS neuroimaging is its wide field of view capable of sensing activity from multiple functionally diverse brain regions, including those that encode different movement effectors, for example, hand and eye. To test this, we decoded intended hand movements to two target directions (reaches to the left/right for monkey P) (Fig. 6). The monkey performed a memory-guided reach task wherein he had to maintain touch on a center dot and touch the peripheral targets during the training (Fig. 6a). In this scenario, we no longer constrained the monkey’s eye position, instead recording hand movements to train the fUS-BMI. After the training period, the monkey controlled the task using the fUS-BMI while keeping his hand on the center fixation cue. Notably, we used the same imaging plane used for eye movement decoding, which contained both LIP (important for eye movements) and MIP (important for reach movements). In an example session using only data from the current session (Fig. 6b), the decoder reached significance after 70 trials and achieved a cumulative decoder accuracy of 61.3%. The decoder predominately guessed left (Fig. 6c). Two foci within the dorsal LIP and scattered voxels throughout area 7a and the temporo-parietal junction (area tpt) contained the most informative voxels for decoding the two reach directions (Fig. 6d).

We evaluated the effect of pretraining the fUS-BMI on an example session (Fig. 6e–g). As with the saccade decoders, pretraining significantly shortened training time. In some cases, pretraining rescued a ‘bad’ model. For example, the example session using only current data (Fig. 6c) displayed a heavy bias toward the left. When we used this same example session to pretrain the fUS-BMI a few days later, the new model made balanced predictions (Fig. 6f). The searchlight analysis for this example session revealed that the same dorsal LIP region from the example session without pretraining contained most of the most informative voxels (Fig. 6g). MIP and area 5 also contained patches of highly informative voxels.

**Fig. 7:**
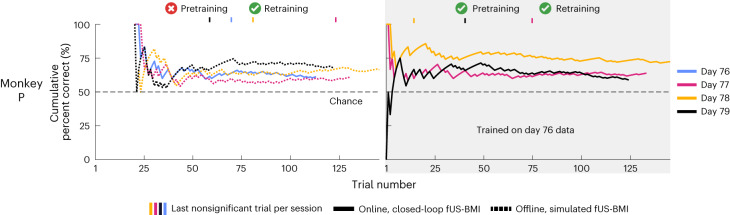
Performance across sessions for decoding two reach directions. Performance across sessions for monkey P. Same format as Fig. 3.

#### Using only data from the current session

The cumulative decoder accuracy reached significance by the end of each session (one real-time and three simulated). The performance reached 65 ± 2% correct and took 67.67 ± 18.77 trials to reach significance (Fig. 7, left).

#### Pretraining with data from a previous session

The cumulative decoder accuracy reached significance by the end of each session (three real-time) (Fig. 7, right). Monkey P’s performance reached 65 ± 4% correct and took 43.67 ± 17.37 trials to reach significance. For two of the three real-time sessions, the number of trials needed to reach significance decreased with pretraining (−2–46 trials faster; 0–16 min faster). There was no statistical difference in performance between the sessions with and without pretraining (paired *t*-test, *P* < 0.05). We also simulated the effects of not using any training data from the current session, that is, using only the pretrained model (Extended Data Fig. 4c). We did not observe a statistically significant difference between the performance (accuracy or number of trials to significant performance), whether current session training data were included or not.

These results are consistent with our previous study’s results^9^ that we can decode not only eye movements, but also reach movements. As with the eye movement decoders, we could pretrain the fUS-BMI using a previous session’s data and reduce, or even eliminate, the need for new training data.

## Discussion

This work demonstrates the successful implementation of a closed-loop, online, ultrasonic BMI, and makes two other key advances that prepare for the next generation of ultrasonic BMIs: decoding more movement directions and stabilizing decoders across more than a month.

### Decoding more movement directions

We successfully decoded eight movement directions in real time, an advance on previous work that decoded two saccade directions and two reach directions using prerecorded data^9^. Specifically, we replicated the two direction results using real-time online data (Figs. 2, 3, 6 and 7) and then extended the decoder to work for eight movement directions (Figs. 4 and 5).

### Stabilizing decoder across time

BMIs using intracortical electrodes, such as Utah arrays, are particularly adept at sensing fast changing (millisecond-scale) neural activity from spatially localized regions (<1 cm) during behavior or stimulation that is correlated to activity in such spatially specific regions, for example, M1 for motor and V1 for vision. Intracortical electrodes, however, struggle to track individual neurons over longer periods of time, for example, between subsequent recording sessions^15,16^. Consequently, decoders are typically retrained every day^15^. A similar neural population identification problem is also present with an ultrasound device, including from shifts in the field of view between experiment sessions. In the current study, we demonstrated an alignment method that stabilizes image-based BMIs across more than a month and decodes from the same neurovascular populations with minimal, if any, retraining. This is a critical development that enables easy alignment of a previous days’ models to a new day’s data and allows decoding to begin with minimal to no new training data. Much effort has focused on ways to recalibrate intracortical BMIs across days that do not require extensive new data^18–23^. Most of these methods require identification of manifolds and/or latent dynamical parameters and collecting new neural and behavioral data to align to these manifolds/parameters. These techniques are, to date, tailored to each research group’s specific applications with varying requirements, such as hyperparameter tuning of the model^23^ or a consistent temporal structure of data^22^. They are also susceptible to changes in function in addition to anatomy. For example, ‘out-of-manifold’ learning/plasticity alters the manifold^24^ in ways that many alignment techniques struggle to address. Finally, some of the algorithms are computationally expensive and/or difficult to implement in online use^22^.

Contrasting these manifold-based methods, our decoder alignment algorithm leverages the intrinsic spatial resolution and field of view provided by fUS neuroimaging to perform decoder stabilization in a way that is intuitive, repeatable and performant. We used a single fUS frame (∼500 ms) to generate an image of the current session’s anatomy and aligned a previous session’s field of view to this single image. Notably, this did not require any additional behavior for the alignment. Because we only relied upon the anatomy, our decoder alignment is robust, can use any off-the-shelf alignment tool and is a valid technique so long as the anatomy and mesoscopic encoding of relevant variables do not change drastically between sessions.

It remains an open question as to how much the precise positioning of the ultrasound transducer during each session matters for decoder performance, especially out-of-plane shifts or rotations. In these current experiments, we used linear decoders that assumed a given image pixel is the same brain voxel across all aligned data sessions. To minimize disruptions to this pixel–voxel relationship, we performed image alignment within the 2D plane. As we could only image a 2D recording plane, we did not correct for any out-of-plane brain shifts between sessions that would have disrupted the pixel–voxel mapping assumption. Future fUS-BMI decoders may benefit from three-dimensional (3D) models of the neurovasculature, such as registering the 2D field of view to a 3D volume^25–27^ to better maintain a consistent pixel–voxel mapping.

### Improving performance

State-of-the-art BMIs using subdural ECoG or intracortical electrodes are currently capable of decoding >15–60 words per min, >29–90 char per min and individual finger movements with high accuracy^2,28–30^ (Supplementary Table 1). Noninvasive scalp EEG is another technology that has been commonly used as a neural basis for control of BMI systems. Performance of modern EEG BMIs varies greatly across users^31^, but using EEG to decode motor imagery or intention can typically yield two degrees of freedom with 70–90% accuracy^32^. This performance is comparable to that described here using fUS. However, fUS performance is rapidly increasing as an evolving neuroimaging technique. We have several ideas about how to improve the fUS-BMI performance.

First, realigning the ultrasound transducer along the intraparietal sulcus axis would allow sampling from a larger portion of LIP and MIP. In this paper, we placed the chamber and probe in a coronal orientation to aid anatomical interpretability. However, most of our imaging plane is not contributing to the decoder performance (Figs. 2c,f, 4c,f and 6d,g). Previous research found that receptive fields are anatomically organized along an anterior–posterior gradient within LIP^17^. By realigning the recording chamber orthogonal to the intraparietal sulcus in future studies, we could sample from a larger anterior–posterior portion of LIP with a more diverse range of directional tunings.

Second, we were limited to 2D planar imaging. The advent of 3D ultrafast volumetric imaging based on matrix or row–column array technologies will be capable of sensing changes in CBV from blood vessels that are currently orthogonal to the imaging plane^26,27^. Additionally, 3D volumetric imaging can fully capture entire functional regions and sense multiple functional regions simultaneously. There are many regions that could fit inside a single 3D probe’s field of view and contribute to a motor BMI, for example, posterior parietal cortex (PPC), primary motor cortex (M1), dorsal premotor cortex (PMd) and supplementary motor area (SMA). These areas encode different aspects of movements including goals, sequences and expected value of actions^33–36^. This is just one example of myriad BMI decoding strategies that will be made possible by synchronous data across brain regions. Currently, high-quality, low-latency real-time 3D fUS imaging is not possible due to bandwidth, memory and compute limitations. However, ongoing advances in hardware and algorithms will likely soon enable 3D fUS-BMI.

Third, another route for improved performance may be to use more advanced decoder models to replace the linear decoders used in this study. Convolutional neural networks are tailor-made for identifying image characteristics and are robust to spatial perturbations common in fUS images, such as brain pulsatility related to breathing or heart rate. Recurrent neural networks and transformers use ‘memory’ processes that may be particularly adept at characterizing the temporal structure of fUS timeseries data. One potential downside of artificial neural networks (ANNs) like these is that they require appreciably more training data. The methods presented here for across-session image alignment allow for previously recorded data to be aggregated and organized into a large data corpus. Such a data corpus may be sufficient to train many ANNs. Aside from the amount of data required to train ANNs, recent work has highlighted additional challenges in training deep learning models for closed-loop motor BMI control^37^, especially avoiding overfitting of the model to the temporal structure in previously recorded data. Although training and inference using ANNs were beyond the scope of the current experiments, this could become an important area of investigation for future, more sophisticated fUS-BMIs^38^.

### Advantages of fUS to existing BMI technologies

fUS has several strengths compared to existing BMI technologies. These include:

1. Large and deep field of view with mesoscopic spatial resolution: the 15.6 MHz ultrasound transducer we used provided a large and deep field of view (12.8 mm × 20 mm) that allows us to reliably record mesoscopic (100 μm) activity from multiple cortical regions simultaneously. The spatial representations of movement variables are often localized to different brain regions. Thus, it is highly advantageous to record from many of these regions in parallel. Additionally, many techniques used for BMI applications are limited to recording from superficial cortex within a few millimeters of the brains surface (Supplementary Table 1). In this study, the most informative voxels for decoding eye movements were located within the mid- to deep LIP, approximately 5–9 mm below the brain surface (Figs. 2c,f and 4c,f), beyond the reach of ECoG, Utah arrays and calcium imaging.
  Previous work found that offline fUS decoding accuracy from PPC decreases rapidly as spatial resolution worsens^9^. This suggests that BMI technologies with macroscopic (≥1 mm) spatial resolution, such as EEG, fNIRS and fMRI, will continue to struggle to effectively decode information that varies within microscopic and mesoscopic neural populations, such as the PPC subregions used in this study.
2. Easy to reposition: with fUS, it is easy to locate and record from specific regions of interest. The ultrasound transducer can be positioned, tested and repositioned multiple times before being locked in place. Invasive electrode arrays are typically inserted only once and are often placed in a suboptimal position due to poor localization or to avoid piercing major vasculature. Implanted electrode arrays are difficult to reposition, as doing so requires additional surgery.
3. Can image through soft tissue: while tissue reactions degrade the performance of subdural and intracortical chronic electrodes^39,40^, fUS can, in principle, operate through the dura indefinitely, enabling chronic imaging over long time periods with minimal, if any, degradation, in signal quality. In a previous monkey study, fUS neuroimaging was able to image through the dura, including the granulation tissue that forms above the dura (approximately several millimeters), with minimal loss in sensitivity across 2.5 years^9^. Distinct from the ability of fUS to image through thickened dura and granulation tissue, future work will be needed to characterize and optimize implanted ultrasound transducer longevity.
4. Easy to align data across sessions: in this Technical Report, we demonstrated a new benefit of fUS, which is decoders that are stable across multiple days or even months. Using conventional image alignment methods, we can align our decoder across different sessions and decode from the start of the session without collecting additional training data (Extended Data Fig. 4). This is similar to the advantage seen with ECoG based BMIs, which can work well across sessions with minimal, if any, recalibration^41–43^. This is likely due, in part, to less representational drift of the mesoscopic neural populations measured by ECoG and fUS compared to the single neurons recorded by intracortical electrodes^44^.

### Disadvantages of fUS to existing BMI technologies

fUS has several weaknesses compared to existing BMI technologies. These include:

1. Temporal resolution: electrophysiological BMIs have superb temporal resolutions (frequently 20–40 KHz) that allow single-spike decoding methods^45–47^. In this study, we used 2-Hz fUS with a real-time latency of approximately 800 ms. fUS is intrinsically limited by the time constant of mesoscopic neurovascular coupling (seconds). Despite this neurovascular response acting as a low pass filter on each voxel’s signal, faster fUS acquisition rates can measure temporal variation across voxels down to 10-ms resolution^48^. Dizeux et al.^48^ performed offline fUS imaging at 100 Hz and tracked rapid (10 ms) propagation of local hemodynamic changes through cortical layers and between functional regions within a single plane of view. Online 100-Hz fUS imaging was not technically feasible with our current equipment and software. However, we performed a lag correlation analysis on previously acquired offline fUS data to approximate the results from 100-Hz fUS imaging (Supplementary Fig. 1). For our seed voxel, correlated activity within superficial cortex and the brain surface preceded the seed voxel activity by approximately 10–50 ms while correlated activity within deeper cortex lagged 10–50 ms behind the seed voxel. This matches with the results from Dizeux et al.^48^ and supports that fUS imaging can detect spatiotemporal patterns at 10-ms time resolution. As the temporal resolution and latency of real-time fUS imaging improves with enhanced hardware and software, tracking the propagation of these rapid hemodynamic signals may enable improved BMI performance and response time. Additionally, for the current study and for many BMI applications, the goals of an action can be extracted despite the slow mesoscopic hemodynamic response and do not require the short latency required for extracting faster signals such as the trajectories of intended movements. Beyond movement, many other signals in the brain may be better suited to the spatial and temporal strengths of fUS, for example, monitoring biomarkers of neuropsychiatric disorders (discussed in detail below).
2. Indirect measure of neural activity: fUS measures local changes in CBV^6^ and is well correlated with local neuronal activity^7,8,49^. As intracranial electrophysiology and calcium optical imaging directly measure activity of individual neurons or small populations of neurons, they are a better control signal for brain–machine interfaces. As acoustic indicators of neural activity are developed^50^, fUS may be able to more directly measure neuronal activity.

### Invasiveness of implant

Our fUS neuroimaging was epidural and required a cranial opening due to significant signal attenuation through bone^11^ (Extended Data Fig. 2). Despite being an intracranial technique, fUS-BMI does not require penetration of the dura mater or cause damage to brain tissue. This decreases the surgical and infection risk compared to subdural ECoG and intracortical electrodes. Penetration of the dura mater increases the risk of serious infections, such as meningitis, cerebritis and empyema^51–53^.

In this study, we used a large cranial opening (∼24 mm × ∼24 mm) made for other experiments that explored multiple brain regions^9^. For future ultrasonic BMIs, a cranial opening would only need to be the size of the ultrasound transducer lens (∼13 mm × 4 mm for the transducer in this study; Extended Data Fig. 2b). Burr holes made by neurosurgeons are frequently 14 mm in diameter, meaning that future versions of this fUS-BMI could be implanted within a single burr hole over the target of interest. Additionally, there is work on performing transcranial fUS neuroimaging using novel ultrasound sequences^54^ or through ultrasound-transparent skull replacement materials^10^.

### Decoding hand versus eye movements

Dorsal and ventral LIP contained the most informative voxels when decoding eye movements (Figs. 2c,f and 4c,f). This is consistent with previous literature that LIP is important for spatially specific oculomotor intention and attention^12^. Dorsal LIP, MIP, area 5 and area 7 contained the most informative voxels during reach movements (Fig. 6d,g). The voxels within the LIP closely match with the most informative voxels from the two-direction saccade decoding, suggesting that our fUS-BMI may be using eye movement plans to build its model of movement direction. The patches of highly informative voxels within MIP and area 5 suggest the fUS-BMI may also be using reach-specific information^13,55,56^. Future experiments will be critical for disentangling the mesoscopic contributions of LIP, MIP, area 5 and other PPC regions for accurate effector predictions with an fUS-BMI. One such experiment would be recording, and ultimately decoding, fUS signals from the PPC as monkeys perform dissociated eye and reach movements^13^. As this fUS-BMI is translated into human applications, these effector-specific signals can also be more cleanly studied by instructing subjects to perform dissociated effector tasks.

In this Technical Report, we demonstrated the utility of the fUS-BMI for motor applications to allow easier comparison with existing BMI technologies. As fUS neuroimaging can record from multiple cortical and subcortical brain regions simultaneously, an exciting future direction will be exploring how fUS-BMI can be used to decode both sensory and motor activity simultaneously in novel BMI paradigms.

### Moving beyond a motor BMI

The vast majority of BMIs have focused on restoring lost motor function in people with paralysis. Recently there has been interest in developing closed-loop BMIs to restore function to other demographics, including patients disabled from neuropsychiatric disorders^1^. Approximately 12% of people worldwide suffer from depression, anxiety or other mood disorders, and first-line treatments only work for ∼33% of patients^57^. Neuropsychiatric BMIs may be a promising avenue for these patients for whom first-line therapies have failed. In one example, a BMI would measure a brain signal that is highly correlated with different mood states. When an aberrant mood state is detected, the BMI could adjust the therapy, such as precisely stimulating specific brain regions^58^.

fUS-BMI may be a platform ideally suited for a neuropsychiatric BMI (Supplementary Table 1). It can record from distributed brain regions; the hemodynamic signal it measures varies on the order of seconds, thus faster than the timescale of mood; it can track the same anatomical volumes across months; and it can be made portable^59^. One possible solution would be to pair fUS imaging with ultrasound neuromodulation (UNM). fUS neuroimaging could track the local network response to the neuromodulation, such that the neuromodulation could not only be precisely adjusted for each patient but for each specific mood aberrance. UNM and fUS imaging may experience interference if used in close space, time and frequency. Frequency-division or time-division multiplexing can likely address this issue without sacrificing overall bandwidth. Current evidence suggests that UNM has effects that can last hours to months after just 0.5–2.0 min of stimulation^60–62^, meaning that fUS would be well-suited to track the local effect of UNM and precisely target the amount of UNM needed to achieve the desired clinical effect.

## Conclusion

The contributions presented here demonstrate the feasibility of an online, closed-loop fUS-BMI. It is still early days with this technology and much work remains to translate this into a clinically viable BMI. However, we believe this work prepares for a new generation of BMIs that are high-resolution, stable across time and scalable to sense activity from large and deep regions of the brain. These advances are a step toward fUS-BMI for a broader range of applications, including restoring function to patients suffering from paralysis or debilitating neuropsychiatric disorders.

## Methods

### Experimental model and subject details

All training, recording, and surgical and animal care procedures were approved by the California Institute of Technology Institutional Animal Care and Use Committee (protocol no. 1256) and complied with the Public Health Service Policy on the Humane Care and Use of Laboratory Animals. We implanted two healthy 14-year-old male rhesus macaque monkeys (*Macaca mulatta*) weighing 14–17 kg.

### General

We used NeuroScan Live software (ART INSERM U1273 and Iconeus) interfaced with MATLAB 2019b (v9.7) (MathWorks) for the real-time fUS-BMI and MATLAB 2021a (v9.10) for all other analyses.

#### Animal preparation and implant

For each monkey, we placed a cranial implant containing a titanium head post and performed a craniectomy positioned over the posterior parietal cortex. The dura underneath the craniectomy was left intact. The craniectomy was covered by a 24 mm × 24 mm (inner dimension) chamber. For each recording session, we used a custom 3D-printed polyetherimide slotted chamber plug that held the ultrasound transducer. This allowed the same anatomical planes to be consistently acquired on different days.

#### Behavioral setup

Monkeys sat in a primate chair facing a monitor or touchscreen. The liquid crystal display monitor was positioned ∼30 cm in front of the monkey. The touchscreen was positioned on each day so that the monkey could reach all the targets on the screen with his fingers but could not rest his palm on the screen. This was ∼20 cm in front of the monkey. Eye position was tracked at 500 Hz using an infrared eye tracker (EyeLink 1000). Touch was tracked using a touchscreen (Elo IntelliTouch). Visual stimuli were presented using custom Python v.2.7 software based on PsychoPy^64^. Eye and hand position were recorded simultaneously with the stimulus and timing information and stored for offline analysis.

### Behavioral tasks

Monkeys performed several different memory-guided movement tasks. In the memory-guided saccade task, monkeys fixated on a center cue for 5 ± 1 s. A peripheral cue appeared for 400 ms in a peripheral location (either chosen from two or eight possible target locations) at 20° eccentricity. The monkey kept fixation on the center cue through a memory period (5 ± 1 s) where the peripheral cue was not visible. The monkey then executed a saccade to the remembered location once the fixation cue was extinguished. If the monkey’s eye position was within a 7° radius of the peripheral target, the target was re-illuminated and stayed on for the duration of the hold period (1.5 ± 0.5 s). The monkey received a liquid reward of 1,000 ms (0.75 ml; dilute juice) for successful task completion. There was an 8 ± 2 s intertrial interval before the next trial began. Fixation, memory and hold periods were subject to ±500 ms timing jitter sampled from a uniform distribution to prevent the monkey from anticipating task state changes.

The memory-guided reach task was similar but, instead of fixation, the monkey used his fingers on a touchscreen. Due to space constraints, eye tracking was not used concurrently with the touchscreen, that is, only hand or eye position was tracked, not both.

For the memory-guided BMI task, the monkey performed the same fixation steps using his eye or hand position, but the movement phase was controlled by the fUS-BMI. Critically, the monkey was trained to not make an eye or hand movement from the center cue until at least the reward was delivered. For this task variant, the monkey received a liquid reward of 1,000 ms (0.75 ml; dilute juice) for successfully maintaining fixation/touch and correct fUS-BMI predictions. The monkey received a 100 ms (0.03 ml; dilute juice) reward for successfully maintaining fixation/touch during incorrect fUS-BMI predictions. This was done to maintain monkey motivation.

### fUS-BMI

#### fUS sequence and recording

During each fUS-BMI session, we placed the ultrasound transducer (128-element miniaturized linear array probe, 15.6-MHz center frequency, 0.1-mm pitch) on the dura with ultrasound gel as a coupling agent (Extended Data Fig. 2b). We consistently positioned the ultrasound transducer across recording sessions using a slotted chamber plug. The imaging field of view was 12.8 mm (width) by 13–20 mm (height) and allowed the simultaneous imaging of multiple cortical regions, including lateral intraparietal area (LIP), medial intraparietal area (MIP), ventral intraparietal area (VIP), area 7 and area 5 (Fig. 1a). In monkey P, we acquired data from the same coronal imaging plane across all experiments. In monkey L, we used two different coronal imaging planes: one for two-target decoding and one for eight-target decoding. The three imaging planes were chosen for good decoding performance in a pilot offline dataset.

We used a programmable high-framerate ultrasound scanner (Vantage 256; Verasonics) to drive the ultrasound transducer and collect pulse-echo radiofrequency data (Extended Data Fig. 2a). We used different plane-wave imaging sequences for real-time and anatomical fUS neuroimaging.

#### Real-time low-latency fUS neuroimaging

We used a custom-built computer running NeuroScan Live (ART INSERM U1273 and Iconeus) attached to the 256-channel Verasonics Vantage ultrasound scanner (Extended Data Fig. 2a). This software implemented a custom plane-wave imaging sequence optimized to deliver power Doppler images in real-time at 2 Hz with minimal latency between ultrasound pulses and power Doppler image formation. The sequence used a pulse-repetition frequency of 5,500 Hz and transmitted plane waves at 11 tilted angles equally spaced from −6° to 6°. These tilted plane waves were compounded at 500 Hz. Power Doppler images were formed from 200 compounded B-mode images (400 ms). To form the power Doppler images, the software used an ultrafast power Doppler sequence with an SVD clutter filter^65^ that discarded the first 30% of components. The resulting power Doppler images were transferred to a MATLAB instance in real-time and used for the fUS-BMI. The prototype 2-Hz real-time fUS system had approximately an 800-ms latency from the end of the ultrasound pulse sequence to arrival of the beamformed fUS image in MATLAB. Each fUS image and associated timing information were saved for post hoc analyses.

#### Anatomical Doppler neuroimaging

At the start of each recording session, we used a custom plane-wave imaging sequence to acquire an anatomical image of the vasculature. We used a pulse-repetition frequency of 7,500 Hz and transmitted plane waves at five angles (−6°, −3°, 0°, 3°, 6°) with three accumulations. We coherently compounded these five angles from three accumulations (15 images) to create one high-contrast ultrasound image. Each high-contrast image was formed in 2 ms, that is, at a 500-Hz framerate. We formed a power Doppler image of the monkey brain using 250 compounded B-mode images collected over 500 ms. We used singular value decomposition to implement a tissue clutter filter and separate blood cell motion from tissue motion^65^.

#### fUS-BMI overview

There were three components to decoding movement intention in real-time: (1) apply preprocessing to a rolling data buffer, (2) train the classifier and (3) decode movement intention in real time using the trained classifier. As described previously^9^, the time for preprocessing, training and decoding was dependent upon several factors, including the number of trials in the training set, CPU load from other applications, the field of view and classifier algorithm (PCA + LDA versus class-wise principal component analysis (cPCA) + LDA). In the worst cases during offline testing, the preprocessing, training and decoder respectively took approximately 10, 500 ms and 60 ms. See ref. ^9^ for further description of the amount of time needed for preprocessing, training and prediction with different sized training sets.

#### Data preprocessing

Before streaming the power Doppler images into the classification algorithm, we applied two preprocessing operations to a rolling 60-frame (30-s) buffer. We first performed a rolling voxel-wise *z*-score over the previous 60 frames (30 s) and then applied a pillbox spatial filter with a radius of 2 pixels to each of the 60 frames in the buffer.

#### Real-time classification

The fUS-BMI made a prediction at the end of the memory period using the preceding 1.5 s of data (three frames) and passed this prediction to the behavioral control system via a TCP-based server (Extended Data Fig. 2c). We used different classification algorithms for fUS-BMI in the two-direction and eight-direction tasks. For decoding two directions of eye or hand movements, we used cPCA and LDA, a method well-suited to classification problems with high dimensional features and low numbers of samples^4,5^. This method is mathematically identical to that used previously for offline decoding of movement intention^9^ but has been optimized for online training and decoding. Briefly, we used cPCA to dimensionally reduce the data while keeping 95% variance of the data. We then used LDA to improve the class separability of the cPCA-transformed data. For more details on the method and implementation, see refs. ^9,66^.

For decoding eight directions of eye movements, we used a multicoder approach where the horizontal (left, center or right) and vertical components (down, center or up) were separately predicted and combined to form the final prediction. As a result of this separate decoding of horizontal and vertical movement components, ‘center’ predictions are possible (horizontal—center and vertical—center) despite this not being one of the eight possible peripheral target locations. We chose this multicoder architecture because we know that similar movement directions will have similar neural responses while movement directions with a large angular separation will have different neural responses. This multicoder thus converts decoding eight separate direction classes into simultaneously decoding two three-class sets that better align with the response properties of PPC. To perform the predictions, we used PCA and LDA. We used the PCA to reduce the dimensionality of the data while keeping 95% of the variance in the data. We then used LDA to predict the most likely direction. We opted for the PCA + LDA method over the cPCA + LDA for eight-direction decoding because we found in offline analyses that the PCA + LDA multicoder outperformed cPCA + LDA for decoding eight movement directions with a limited number of training trials.

#### Real-time training of model

We retrained the fUS-BMI classifier during the intertrial interval (without stopping the experiment) every time the training set was updated. For the real-time experiments, the data recorded during a successful trial were automatically added to the training set. During the initial training phase, successful trials were defined as the monkey performing the movement to the correct target and receiving his liquid reward. Once in BMI mode, successful trials were defined as a correct prediction plus the monkey maintaining fixation until reward delivery.

For experiments that used a model trained on data from a previous session, we used data from all valid trials from the previous session upon initialization of the fUS-BMI. A valid trial was defined as any trial that reached the prediction phase, regardless of whether the correct class was predicted. The classifier then retrained after the addition of each successful trial to the training set during the current session.

#### Post hoc experiments

These experiments analyzed the effect of using only data from a single session on decoder performance. We simulated an online scenario where we trained and/or decoded on each attempted trial in order. We considered all trials where the monkey received a reward as successful and retrained after each trial.

#### Connection with the behavioral system

We designed a threaded TCP server in Python v.2.7 to receive, parse and send information between the computer running the PsychoPy behavior software and the real-time fUS-BMI computer (Extended Data Fig. 2c). Upon queries from the fUS-BMI computer, this server transferred task information, including task timing and actual movement direction, to the real-time ultrasound system. The client–server architecture was specifically designed to prevent data leaks: the actual movement direction was never transmitted to the fUS-BMI until after a successful trial had ended. The TCP server also received the fUS-BMI prediction and passed it to the PsychoPy software when queried. The average server write–read–parse time was 31 ± 1 (mean ± s.d.) ms during offline testing between two desktop computers (Windows) on a local area network.

### Across-session alignment

At the beginning of each experimental session, we acquired an anatomical image showing the vasculature within the imaging field of view. For sessions where we used previous data as the initial training set for the fUS-BMI, we then performed a semiautomated intensity-based rigid-body alignment between the new anatomical image and the anatomical image acquired in a previous session. We used the MATLAB ‘imregtform’ function with the mean square error metric and a regular step gradient descent optimizer to generate an initial automated alignment of the previous anatomical image to the new anatomical image. If the automated alignment had misaligned the two images, the software prompted the proctor to manually shift and rotate the previous session’s anatomical image using a custom MATLAB graphical user interface. We then applied the final rigid-body transform to the training data from the previous session, thus aligning the previous session with the new session.

We chose rigid-body alignment and transformation over Procrustes alignment and transformations because the brain size did not vary across sessions. Although we did observe brain pulsatility between sequential fUS frames (due in part to heart rate and breathing), this pulsatility did not affect the rigid-body alignment accuracy. Additionally, the amount of brain expansion and/or shrinkage from pulsatility varied across the image. The brain surface would move up and down, but brain tissue within a few millimeters of the brain surface would be stable. Uniformly scaling the image (such as via a Procrustes transform) would not fix this issue but rather introduce new alignment issues.

### Quantification, statistical analysis and reproducibility

Unless reported otherwise, summary statistics are reported as *XX* ± *XX* are mean ± s.e.m.

#### Accuracy metrics

We used cumulative percent correct and mean angular error to assess the performance of the fUS-BMI. All accuracy metrics reported reflect the test performance rather than performance on the training set, that is, we trained the model using the training set and then tested on data not within the training set.

$${\rm{Cumulative}}\; {\rm{percent}}\; {\rm{correct}}=\frac{\text{No. of correct}\,\text{predictions}}{\text{No. of total}\,\text{predictions}}$$

$$\begin{array}{l}{\rm{Mean}}\,{\rm{angular}}\,{\rm{error}}\\=\frac{1}{n}\mathop{\sum }\limits_{i=1}^{n}|{\rm{angular}}\,{\rm{error}}|,{\rm{where}}\,n\,{\rm{is}}\,{\rm{the}}\,{\rm{number}}\,{\rm{of}}\,{\rm{predictions}}\end{array}$$

The chance envelope for the cumulative percent correct was calculated using the binomial distribution and the number of total predictions. Accuracies above or below the chance envelope were significant at *α* = 0.05. Assumptions for using a binomial test were satisfied including each trial having only two possible outcomes (success/failure), the probability of success was the same for all trials (1/*n* where *n* is the number of possible movement directions), and each trial being independent. The chance envelope for the mean angular error was calculated using a permutation test with 10^5^ replicates. For each replicate, we randomly sampled *n* times from a uniform distribution of the eight possible directions, where *n* was the number of predictions in the entire session. This generated a null distribution of chance level decoding as a function of the number of predictions. We then found the 5th and 95th quantiles of the null distribution to generate the chance envelope as a function of the number of predictions. For the paired *t*-tests comparing performance between the same sessions with or without pretraining or retraining, the data distribution was assumed to be normal, but this was not formally tested.

#### Post hoc simulated session

We used the recorded real-time fUS images to simulate the effects of different parameters on fUS-BMI performance, such as using only current session data without pretraining. To do this, we streamed prerecorded fUS images and behavioral data, frame-by-frame, to the same fUS-BMI function used for closed-loop, online fUS-BMI. To dynamically build the training set, we added all trials reaching the end of the memory phase regardless of whether the offline fUS-BMI predicted the correct movement direction. This was done because the high possible error rate from bad predictions meant that building the training set from only correctly predicted trials could lead to imbalanced samples across conditions (directions) and possibly contain insufficient trials to train the model. Zero correct predictions for certain directions could prevent the model from ever predicting that direction.

#### Searchlight analysis

We defined a circular region of interest (ROI; 200-μm radius) and moved it sequentially across all voxels in the imaging field of view. For each ROI, we performed offline decoding with 10-fold cross-validation using either the cPCA + LDA (two-directions) or PCA + LDA (eight-directions) algorithm where we only used the voxels fully contained with each ROI. We assigned the mean performance across the cross-validation folds to the center voxel of the ROI. To visualize the results, we overlaid the performance (mean angular error or accuracy) of the 10% most significant voxels on the anatomical vascular map from the session.

#### Reproducibility

We collected data across 24 sessions (Monkey L, 11 sessions; monkey P, 13 sessions; Extended Data Table 1). These sessions were split across three different sets of experiments with 10 sessions of the two-target saccade experiment, 10 sessions of the eight-target saccade experiment and four sessions of the two-target reach experiment. Within each session, the order of different target directions was pseudo-randomized based on a Latin square design. No statistical method was used to predetermine sample size for number of sessions. No statistical method was used to predetermine sample size for number of monkeys but our sample sizes are similar to those reported in previous publications^9,18,20^. No sessions or data points were excluded from the analyses. Data collection and analysis were not performed blind to the conditions of the experiments.

### Reporting summary

Further information on research design is available in the Nature Portfolio Reporting Summary linked to this article.

## Online content

Any methods, additional references, Nature Portfolio reporting summaries, source data, extended data, supplementary information, acknowledgements, peer review information; details of author contributions and competing interests; and statements of data and code availability are available at 10.1038/s41593-023-01500-7.

### Supplementary information

Supplementary Information Supplementary Fig. 1 and Table 1.

Reporting Summary

## Figures and Tables

**Extended Data Table 1:**
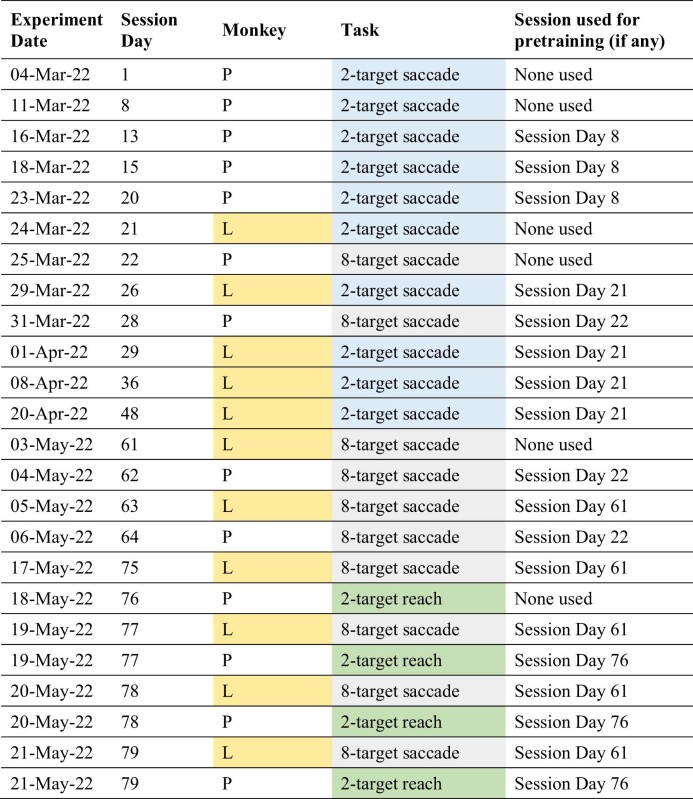
Session record

A record of all experimental sessions, including the experiment date, monkey participant, task, and what session was used for pretraining. Session day is relative to the first session day.

## Data Availability

Key data used in this paper are archived at 10.22002/pa710-cdn95.

## Code availability

Code used to generate key figures and results is available at https://github.com/wsgriggs2/rt_fUS_BMI and archived at 10.5281/zenodo.8414598.

## References

[1] Shanechi MM (2019). Brain–machine interfaces from motor to mood. Nat. Neurosci..

[2] Willett, F. R. et al. A high-performance speech neuroprosthesis. *Nature* **620**, 1031–1036 (2023). 10.1038/s41586-023-06377-x PMC10468393 37612500

[3] Collinger JL (2013). High-performance neuroprosthetic control by an individual with tetraplegia. Lancet.

[4] Sorger B, Reithler J, Dahmen B, Goebel R (2012). A real-time fMRI-based spelling device immediately enabling robust motor-independent communication. Curr. Biol..

[5] Yoo, S.-S. et al. Brain–computer interface using fMRI: spatial navigation by thoughts. *NeuroReport* **15**, 1591–1595 (2004). 10.1097/01.wnr.0000133296.39160.fe 15232289

[6] Macé E (2011). Functional ultrasound imaging of the brain. Nat. Methods.

[7] Claron J (2023). Co-variations of cerebral blood volume and single neurons discharge during resting state and visual cognitive tasks in non-human primates. Cell Rep..

[8] Nunez-Elizalde, A. O. et al. Neural correlates of blood flow measured by ultrasound. *Neuron* **110**, 1631–1640 (2022). 10.1016/j.neuron.2022.02.012 PMC9235295 35278361

[9] Norman, S. L. et al. Single-trial decoding of movement intentions using functional ultrasound neuroimaging. *Neuron* **109**, 1554–1566 (2021). 10.1016/j.neuron.2021.03.003 PMC8105283 33756104

[10] Rabut, C. et al. A window to the brain: ultrasound imaging of human neural activity through a permanent acoustic window. Preprint at *bioRxiv*10.1101/2023.06.14.544094 (2023).

[11] Pinton G (2012). Attenuation, scattering, and absorption of ultrasound in the skull bone. Med. Phys..

[12] Andersen RA, Cui H (2009). Intention, action planning, and decision making in parietal-frontal circuits. Neuron.

[13] Snyder LH, Batista AP, Andersen RA (1997). Coding of intention in the posterior parietal cortex. Nature.

[14] Christopoulos VN, Kagan I, Andersen RA (2018). Lateral intraparietal area (LIP) is largely effector-specific in free-choice decisions. Sci. Rep..

[15] Downey JE, Schwed N, Chase SM, Schwartz AB, Collinger JL (2018). Intracortical recording stability in human brain-computer interface users. J. Neural Eng..

[16] Santhanam G (2007). HermesB: a continuous neural recording system for freely behaving primates. IEEE Trans. Biomed. Eng..

[17] Patel, G. H., Kaplan, D. M. & Snyder, L. H. Topographic organization in the brain: searching for general principles. *Trends Cogn. Sci.* **18**, 351–363 (2014). 10.1016/j.tics.2014.03.008 PMC4074559 24862252

[18] Sussillo D, Stavisky SD, Kao JC, Ryu SI, Shenoy KV (2016). Making brain–machine interfaces robust to future neural variability. Nat. Commun..

[19] Pandarinath C (2018). Latent factors and dynamics in motor cortex and their application to brain–machine interfaces. J. Neurosci..

[20] Degenhart AD (2020). Stabilization of a brain–computer interface via the alignment of low-dimensional spaces of neural activity. Nat. Biomed. Eng..

[21] Wilson, G. H. et al. Long-term unsupervised recalibration of cursor BCIs. Preprint at *bioRxiv*10.1101/2023.02.03.527022 (2023).

[22] Karpowicz, B. M. et al. Stabilizing brain-computer interfaces through alignment of latent dynamics. Preprint at *bioRxiv*10.1101/2022.04.06.487388 (2022). 10.1038/s41467-025-59652-y PMC12089531 40389429

[23] Ma, X. et al. Using adversarial networks to extend brain computer interface decoding accuracy over time. *eLife* **12**, e84296 (2023). 10.7554/eLife.84296 PMC10446822 37610305

[24] Oby ER (2019). New neural activity patterns emerge with long-term learning. Proc. Natl Acad. Sci. USA.

[25] Demené C (2016). 4D microvascular imaging based on ultrafast Doppler tomography. NeuroImage.

[26] Rabut C (2019). 4D functional ultrasound imaging of whole-brain activity in rodents. Nat. Methods.

[27] Brunner C (2020). A platform for brain-wide volumetric functional ultrasound imaging and analysis of circuit dynamics in awake mice. Neuron.

[28] Metzger SL (2022). Generalizable spelling using a speech neuroprosthesis in an individual with severe limb and vocal paralysis. Nat. Commun..

[29] Willett FR, Avansino DT, Hochberg LR, Henderson JM, Shenoy KV (2021). High-performance brain-to-text communication via handwriting. Nature.

[30] Guan, C. et al. Compositional coding of individual finger movements in human posterior parietal cortex and motor cortex enables ten-finger decoding. Preprint at *medRxiv*10.1101/2022.12.07.22283227 (2022).

[31] Ahn, M. & Jun, S. C. Performance variation in motor imagery brain–computer interface: a brief review. *J. Neurosci. Methods* **243**, 103–110 (2015). 10.1016/j.jneumeth.2015.01.033 25668430

[32] Huang D, Lin P, Fei D-Y, Chen X, Bai O (2009). Decoding human motor activity from EEG single trials for a discrete two-dimensional cursor control. J. Neural Eng..

[33] Taylor DM, Tillery SIH, Schwartz AB (2002). Direct cortical control of 3D neuroprosthetic devices. Science.

[34] Ohbayashi M, Picard N, Strick PL (2016). Inactivation of the dorsal premotor area disrupts internally generated, but not visually guided, sequential movements. J. Neurosci..

[35] Côté SL, Elgbeili G, Quessy S, Dancause N (2020). Modulatory effects of the supplementary motor area on primary motor cortex outputs. J. Neurophysiol..

[36] Platt ML, Glimcher PW (1999). Neural correlates of decision variables in parietal cortex. Nature.

[37] Deo, D. R. et al. Translating deep learning to neuroprosthetic control. Preprint at *bioRxiv*10.1101/2023.04.21.537581 (2023).

[38] Berthon B, Bergel A, Matei M, Tanter M (2023). Decoding behavior from global cerebrovascular activity using neural networks. Sci. Rep..

[39] Szymanski LJ (2021). Neuropathological effects of chronically implanted, intracortical microelectrodes in a tetraplegic patient. J. Neural Eng..

[40] Degenhart AD (2016). Histological evaluation of a chronically-implanted electrocorticographic electrode grid in a non-human primate. J. Neural Eng..

[41] Moses DA (2021). Neuroprosthesis for decoding speech in a paralyzed person with anarthria. N. Engl. J. Med..

[42] Chao ZC, Nagasaka Y, Fujii N (2010). Long-term asynchronous decoding of arm motion using electrocorticographic signals in monkeys. Front Neuroeng..

[43] Silversmith DB (2021). Plug-and-play control of a brain–computer interface through neural map stabilization. Nat. Biotechnol..

[44] Clopath C, Bonhoeffer T, Hübener M, Rose T (2017). Variance and invariance of neuronal long-term representations. Philos. Trans. R. Soc. B: Biol. Sci..

[45] Shanechi MM (2017). Rapid control and feedback rates enhance neuroprosthetic control. Nat. Commun..

[46] Shanechi MM (2013). A real-time brain-machine interface combining motor target and trajectory intent using an optimal feedback control design. PLoS ONE.

[47] Shanechi MM, Orsborn AL, Carmena JM (2016). Robust brain-machine interface design using optimal feedback control modeling and adaptive point process filtering. PLoS Comput. Biol..

[48] Dizeux A (2019). Functional ultrasound imaging of the brain reveals propagation of task-related brain activity in behaving primates. Nat. Commun..

[49] Aydin A-K (2020). Transfer functions linking neural calcium to single voxel functional ultrasound signal. Nat. Commun..

[50] Shapiro MG (2014). Biogenic gas nanostructures as ultrasonic molecular reporters. Nat. Nanotech.

[51] van de Beek D, Drake JM, Tunkel AR (2010). Nosocomial bacterial meningitis. N. Engl. J. Med..

[52] McClelland S, Hall WA (2007). Postoperative central nervous system infection: incidence and associated factors in 2111 neurosurgical procedures. Clin. Infect. Dis..

[53] Korinek, A.-M. et al. Risk Factors for adult nosocomial meningitis after craniotomy: role of antibiotic prophylaxis. *Neurosurgery* **59**, 126–133 (2006). 10.1227/01.NEU.0000220477.47323.92 16823308

[54] Vienneau, E. P. & Byram, B. C. A coded excitation framework for high SNR transcranial ultrasound imaging. *IEEE Trans. Med. Imaging* **42**, 2886–2898 (2023). 10.1109/TMI.2023.3269022 PMC10691235 37079411

[55] Lacquaniti, F., Guigon, E., Bianchi, L., Ferraina, S. & Caminiti, R. Representing spatial information for limb movement: role of area 5 in the monkey. *Cereb. Cortex* **5**, 391–409 (1995). 10.1093/cercor/5.5.391 8547787

[56] Chang SWC, Papadimitriou C, Snyder LH (2009). Using a compound gain field to compute a reach plan. Neuron.

[57] Rush, A. J. Unipolar major depression in adults: choosing initial treatment. *UpToDate* www.uptodate.com/contents/unipolar-major-depression-in-adults-choosing-initial-treatment/print (2023).

[58] Scangos KW (2021). Closed-loop neuromodulation in an individual with treatment-resistant depression. Nat. Med..

[59] Deffieux, T., Demene, C., Pernot, M. & Tanter, M. Functional ultrasound neuroimaging: a review of the preclinical and clinical state of the art. *Curr. Opin. Neurobiol.* **50**, 128–135 (2018). 10.1016/j.conb.2018.02.001 29477979

[60] Sanguinetti JL (2020). Transcranial focused ultrasound to the right prefrontal cortex improves mood and alters functional connectivity in humans. Front. Hum. Neurosci..

[61] Matt E (2022). First evidence of long-term effects of transcranial pulse stimulation (TPS) on the human brain. J. Transl. Med..

[62] Verhagen L (2019). Offline impact of transcranial focused ultrasound on cortical activation in primates. eLife.

[63] Saleem, K. S. *A Combined MRI and Histology Atlas of the Rhesus Monkey Brain in Stereotaxic Coordinates* (Academic Press, 2012).

[64] Peirce, J. W. PsychoPy—psychophysics software in Python. *J. Neurosci. Methods* **162**, 8–13 (2007). 10.1016/j.jneumeth.2006.11.017 PMC2018741 17254636

[65] Demené C (2015). Spatiotemporal clutter filtering of ultrafast ultrasound data highly increases Doppler and fUltrasound sensitivity. IEEE Trans. Med. Imaging.

[66] Das K, Nenadic Z (2009). An efficient discriminant-based solution for small sample size problem. Pattern Recognit..

