# Supplementary material for: Decoding motor plans using a closed-loop ultrasonic brain–machine interface
Source: Nat Neurosci. 2023 Nov 30;27(1):196–207. doi: 10.1038/s41593-023-01500-7 (PMC10774125; doi:10.1038/s41593-023-01500-7)
Supplement: Supplementary file 1 — Supplementary Fig. 1 and Table 1. [file 41593_2023_1500_MOESM1_ESM.pdf]

nature neuroscience

Technical Report https://doi.org/10.1038/s41593-023-01500-7

Decoding motor plans using a closed-loop ultrasonic brain–machine interface

In the format provided by the authors and unedited

Supplementary Data Table 1: Comparison of real-time BMI technologies (typical values)

Method	Example real-time performance	Invasiveness	Temporal resolution	Spatial resolution	Recording depth	Spatial coverage	Device durability	Across-session stability	Portability	Refs.
**fMRI**	1 char/min (82% accuracy)	Non-invasive	0.5-1 Hz	1.5-4 mm isotropic voxels	Whole brain	Whole-brain 3D	No data	Stable across years	Non-portable	1–11
**EEG**	50 char/min (91% accuracy)<br>2-dimensional cursor control (70-90% accuracy)	Non-invasive	250-1000 Hz	1 – 10 cm	Surface cortex<br>Some ability to source localize subcortical structures	Whole-brain 2D	No data	No data	Portable	12–19
**fNIRS**	3 classes (84% accuracy)	Non-invasive	7-10 Hz	2-3 cm	2 cm	Whole-brain 2D	No data	No data	Portable	20–26
**2D fUS**	2 classes (80% accuracy)<br>8 classes (36% accuracy)	Epidural	2-100 Hz	50 -500 μm	2 - 5 cm	12.8-25.6 mm x 400-800 μm	No data	Stable	Semi-portable	27–31
**ECoG**	29 char/min;<br>15 words/min<br>8 degrees of freedom (64-99% accuracy)	Epidural subdural	20-40 kHz	0.5-4 mm pitch	Surface cortex	~5-10 cm x 3-5 cm	Years	Stable	Semi-portable	32–36
**Calcium imaging**	2 class (87% accuracy)<br>4 class (70% accuracy)	Subdural	30 Hz	0.2-1 μm	150-350 μm	~600 x 600 μm	No data	No data	Semi-portable	37
**Utah Array**	90 char/min;<br>62 words/min<br>10 degrees of freedom (70-78% success rate)	Intracortical	20-40 kHz	Single cell isolation, electrodes spaced 400 μm apart	1-1.5 mm	4.4 x 4.2 mm	Years	Needs new training data	Semi-portable	38–44

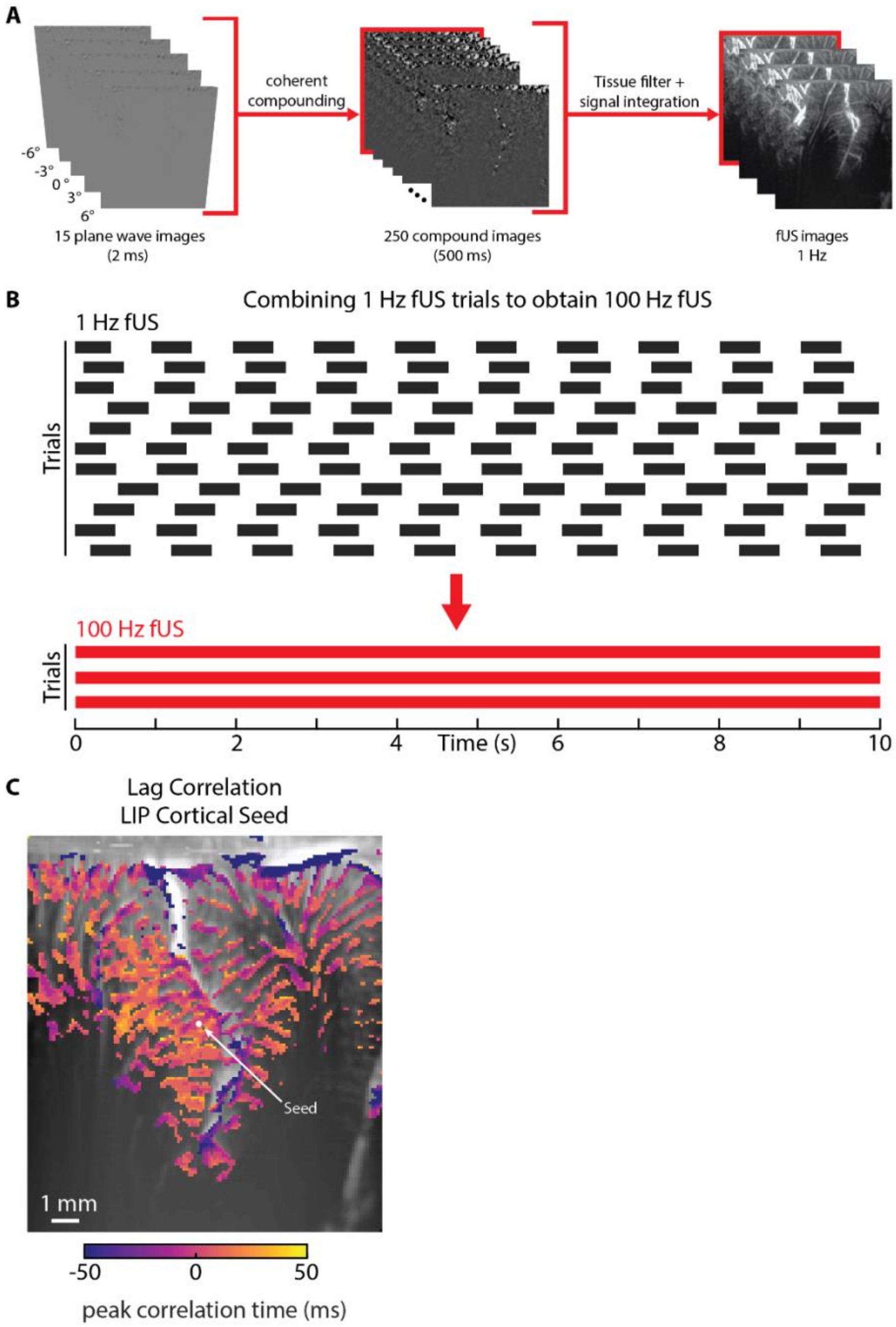

Supplementary Fig. 1 – Combining offline 1 Hz fUS trials into new 100 Hz fUS trials
(A) Acquisition pipeline of offline 1 Hz fUS data. Ultrafast plane wave ultrasound images are acquired at 5 angles with 3 accumulations for a total of 2 ms. These plane-wave images are coherently compounded into a single image. These compounded images are generated at 500 Hz. A series of compounded images are formed into a single Power Doppler image. The final images are 1 Hz due to hardware and software limitations requiring ~500 ms of downtime to transfer and save the compounded images.
(B) We recorded 1 Hz fUS data while our monkeys performed the 8-direction memory-guided saccade task. Multiple 1 Hz fUS trials for the same movement directions were combined to form new 100 Hz fUS trials. To form the 100 Hz fUS trials, we beamformed 100 compound images (200 ms of data) in a 10 ms sliding window. This generated discrete chunks of 100 Hz fUS data. We then aligned these discrete chunks of 100 Hz fUS data to the behavior and combined the chunks of 100 Hz fUS across trials to generate new trials with complete time coverage.
(C) Lag correlation for seed voxel within LIP cortex. Time of peak correlation is displayed. Only peak correlations > 0.2 are displayed.

REFERENCES

1. Sorger, B., Reithler, J., Dahmen, B. & Goebel, R. A Real-Time fMRI-Based Spelling Device Immediately Enabling Robust Motor-Independent Communication. *Current Biology* **22**, 1333–1338 (2012).
2. Sorger, B. & Goebel, R. Chapter 21 - Real-time fMRI for brain-computer interfacing. in *Handbook of Clinical Neurology* (eds. Ramsey, N. F. & Millán, J. del R.) vol. 168 289–302 (Elsevier, 2020).
3. Cortese, A. *et al.* The DecNef collection, fMRI data from closed-loop decoded neurofeedback experiments. *Sci Data* **8**, 65 (2021).
4. Kaas, A., Goebel, R., Valente, G. & Sorger, B. Topographic Somatosensory Imagery for Real-Time fMRI Brain-Computer Interfacing. *Front Hum Neurosci* **13**, 427 (2019).
5. Yoo, S.-S. *et al.* Brain–computer interface using fMRI: spatial navigation by thoughts. *NeuroReport* **15**, 1591 (2004).
6. Lee, J.-H., Ryu, J., Jolesz, F. A., Cho, Z.-H. & Yoo, S.-S. Brain–machine interface via real-time fMRI: Preliminary study on thought-controlled robotic arm. *Neuroscience Letters* **450**, 1–6 (2009).
7. LaConte, S. M. Decoding fMRI brain states in real-time. *NeuroImage* **56**, 440–454 (2011).
8. Andersson, P. *et al.* Real-Time Decoding of Brain Responses to Visuospatial Attention Using 7T fMRI. *PLOS ONE* **6**, e27638 (2011).
9. Andersson, P., Ramsey, N. F., Pluim, J. P. W. & Viergever, M. A. BCI control using 4 direction spatial visual attention and real-time fMRI at 7T. in *2010 Annual International Conference of the IEEE Engineering in Medicine and Biology* 4221–4225 (IEEE, 2010). doi:10.1109/IEMBS.2010.5627372.
10. Eklund, A., Andersson, M., Ohlsson, H., Ynnerman, A. & Knutsson, H. A Brain Computer Interface for Communication Using Real-Time fMRI. in *2010 20th International Conference on Pattern Recognition* 3665–3669 (2010). doi:10.1109/ICPR.2010.894.
11. Naci, L., Cusack, R., Jia, V. Z. & Owen, A. M. The Brain's Silent Messenger: Using Selective Attention to Decode Human Thought for Brain-Based Communication. *J. Neurosci.* **33**, 9385–9393 (2013).
12. Spüler, M., Rosenstiel, W. & Bogdan, M. Online Adaptation of a c-VEP Brain-Computer Interface(BCI) Based on Error-Related Potentials and Unsupervised Learning. *PLoS One* **7**, e51077 (2012).
13. Huang, D., Lin, P., Fei, D.-Y., Chen, X. & Bai, O. Decoding human motor activity from EEG single trials for a discrete two-dimensional cursor control. *J. Neural Eng.* **6**, 046005 (2009).
14. Orban, M., Elsamanty, M., Guo, K., Zhang, S. & Yang, H. A Review of Brain Activity and EEG-Based Brain–Computer Interfaces for Rehabilitation Application. *Bioengineering (Basel)* **9**, 768 (2022).
15. Gevins, A., Smith, M. E., McEvoy, L. K., Leong, H. & Le, J. Electroencephalographic imaging of higher brain function. *Phil. Trans. R. Soc. Lond. B* **354**, 1125–1134 (1999).
16. Nagel, S. & Spüler, M. World's fastest brain-computer interface: Combining EEG2Code with deep learning. *PLOS ONE* **14**, e0221909 (2019).
17. Krishnaswamy, P. *et al.* Sparsity enables estimation of both subcortical and cortical activity from MEG and EEG. *Proceedings of the National Academy of Sciences* **114**, E10465–E10474 (2017).
18. Seeber, M. *et al.* Subcortical electrophysiological activity is detectable with high-density EEG source imaging. *Nat Commun* **10**, 753 (2019).
19. Chen, X. *et al.* High-speed spelling with a noninvasive brain–computer interface. *Proceedings of the National Academy of Sciences* **112**, E6058–E6067 (2015).
20. Sakai, J. Functional near-infrared spectroscopy reveals brain activity on the move. *Proc Natl Acad Sci USA* **119**, e2208729119 (2022).
21. Hong, K.-S., Ghafoor, U. & Khan, M. J. Brain–machine interfaces using functional near-infrared spectroscopy: a review. *Artif Life Robotics* **25**, 204–218 (2020).
22. Rolfe, P. In Vivo Near-Infrared Spectroscopy. *Annual Review of Biomedical Engineering* **2**, 715–754 (2000).
23. Coyle, S. M., Ward, T. E. & Markham, C. M. Brain computer interface using a simplified functional near-infrared spectroscopy system. *J Neural Eng* **4**, 219–226 (2007).
24. Sitaram, R. *et al.* Temporal classification of multichannel near-infrared spectroscopy signals of motor imagery for developing a brain–computer interface. *NeuroImage* **34**, 1416–1427 (2007).
25. Pinti, P. *et al.* The present and future use of functional near-infrared spectroscopy (fNIRS) for cognitive neuroscience. *Ann N Y Acad Sci* **1464**, 5–29 (2020).

26. Naseer, N. & Hong, K.-S. Decoding Answers to Four-Choice Questions Using Functional near Infrared Spectroscopy. *Journal of Near Infrared Spectroscopy* **23**, 23–31 (2015).
27. Dizeux, A. *et al.* Functional ultrasound imaging of the brain reveals propagation of task-related brain activity in behaving primates. *Nat Commun* **10**, 1400 (2019).
28. Norman, S. L. *et al.* Single-trial decoding of movement intentions using functional ultrasound neuroimaging. *Neuron* (2021) doi:10.1016/j.neuron.2021.03.003.
29. Berthon, B., Bergel, A., Matei, M. & Tanter, M. Decoding behavior from global cerebrovascular activity using neural networks. *Sci Rep* **13**, 3541 (2023).
30. Montaldo, G., Urban, A. & Macé, E. Functional ultrasound neuroimaging. *Ann Rev Neurosci* **45**, 491–513 (2022).
31. Baranger, J. *et al.* Bedside functional monitoring of the dynamic brain connectivity in human neonates. *Nat Commun* **12**, 1080 (2021).
32. Moses, D. A. *et al.* Neuroprosthesis for decoding speech in a paralyzed person with anarthria. *N Engl J Med* **385**, 217–227 (2021).
33. Metzger, S. L. *et al.* Generalizable spelling using a speech neuroprosthesis in an individual with severe limb and vocal paralysis. *Nat Commun* **13**, 6510 (2022).
34. Benabid, A. L. *et al.* An exoskeleton controlled by an epidural wireless brain-machine interface in a tetraplegic patient: A proof-of-concept demonstration. *Lancet Neurol* **18**, 1112–1122 (2019).
35. Degenhart, A. D. *et al.* Stabilization of a brain–computer interface via the alignment of low-dimensional spaces of neural activity. *Nat Biomed Eng* **4**, 672–685 (2020).
36. Moly, A. *et al.* An adaptive closed-loop ECoG decoder for long-term and stable bimanual control of an exoskeleton by a tetraplegic. Preprint at http://arxiv.org/abs/2201.10449 (2022).
37. Trautmann, E. M. *et al.* Dendritic calcium signals in rhesus macaque motor cortex drive an optical brain-computer interface. *Nat Commun* **12**, 3689 (2021).
38. Willett, F. *et al.* A high-performance speech neuroprosthesis. *bioRxiv* 2023.01.21.524489 (2023) doi:10.1101/2023.01.21.524489.
39. Blackrock Microsystems. Utah Array Surgical Manual - Research. (2020).
40. Willett, F. R., Avansino, D. T., Hochberg, L. R., Henderson, J. M. & Shenoy, K. V. High-performance brain-to-text communication via handwriting. *Nature* **593**, 249–254 (2021).
41. Bullard, A. J., Hutchison, B. C., Lee, J., Chestek, C. A. & Patil, P. G. Estimating risk for future intracranial, fully implanted, modular neuroprosthetic systems: a systematic review of hardware complications in clinical deep brain stimulation and experimental human intracortical arrays. *Neuromodulation* **23**, 411–426 (2020).
42. Sponheim, C. *et al.* Longevity and reliability of chronic unit recordings using the Utah, intracortical multi-electrode arrays. *J. Neural Eng.* **18**, 066044 (2021).
43. Guan, C. *et al.* Stability of motor representations after paralysis. *eLife* **11**, e74478 (2022).
44. Flesher, S. N. *et al.* A brain-computer interface that evokes tactile sensations improves robotic arm control. *Science* **372**, 831–836 (2021).
